# Sample size and power calculations for detecting changes in malaria transmission using antibody seroconversion rate

**DOI:** 10.1186/s12936-015-1050-3

**Published:** 2015-12-30

**Authors:** Nuno Sepúlveda, Carlos Daniel Paulino, Chris Drakeley

**Affiliations:** London School of Hygiene and Tropical Medicine, Keppel Street, London, WC1E 7HT UK; Center of Statistics and Applications of University of Lisbon, Faculdade de Ciências da Universidade de Lisboa, Bloco C6-Piso 4, 1749-1016 Lisbon, Portugal; Instituto Superior Técnico, Universidade Técnica de Lisboa, Avenida Rovisco Pais, 1049-001 Lisbonn, Portugal

**Keywords:** Intervention, Malaria transmission, Bias, Precision, Sample size

## Abstract

**Background:**

Several studies have highlighted the use of serological data in detecting a reduction in malaria transmission intensity. These studies have typically used serology as an adjunct measure and no formal examination of sample size calculations for this approach has been conducted.

**Methods:**

A sample size calculator is proposed for cross-sectional surveys using data simulation from a reverse catalytic model assuming a reduction in seroconversion rate (SCR) at a given change point before sampling. This calculator is based on logistic approximations for the underlying power curves to detect a reduction in SCR in relation to the hypothesis of a stable SCR for the same data. Sample sizes are illustrated for a hypothetical cross-sectional survey from an African population assuming a known or unknown change point.

**Results:**

Overall, data simulation demonstrates that power is strongly affected by assuming a known or unknown change point. Small sample sizes are sufficient to detect strong reductions in SCR, but invariantly lead to poor precision of estimates for current SCR. In this situation, sample size is better determined by controlling the precision of SCR estimates. Conversely larger sample sizes are required for detecting more subtle reductions in malaria transmission but those invariantly increase precision whilst reducing putative estimation bias.

**Conclusions:**

The proposed sample size calculator, although based on data simulation, shows promise of being easily applicable to a range of populations and survey types. Since the change point is a major source of uncertainty, obtaining or assuming prior information about this parameter might reduce both the sample size and the chance of generating biased SCR estimates.

**Electronic supplementary material:**

The online version of this article (doi:10.1186/s12936-015-1050-3) contains supplementary material, which is available to authorized users.

## Background

The global decline of malaria burden has brought new challenges to disease control and elimination [1]. These challenges encompass problems related to parasite rate (PR) estimation in detecting low parasitaemia or sub-microscopic infections [2–4] and potentially prohibitive large sample sizes for PR to be epidemiologically informative. In low transmission settings, alternative malariometrics, such as anti-malarial antibody seroprevalence (SP) and seroconversion rate (SCR) have been proposed to overcome some shortcomings of other measures [5]. In practice, SP is statistically defined as the proportion of antibody-positive individuals and reflects antibody responses induced by current and possibly historic infections. Two recent studies highlighted the potential of using SP to discriminate sites with different *Plasmodium falciparum* endemicity levels that otherwise would appear to be similar in terms of parasite rate [6, 7]. SCR is the frequency per unit of time (e.g., year) by which seronegative individuals become seropositive. This parameter, related to the underlying force-of-infection, is typically assessed via cross-sectional data where SP as function of age of the individual is described by a given stochastic model. The reverse catalytic model is the most popular choice for data analysis and based on the simple notion that individuals randomly transit between seronegativity and seropositivity with specific transition rates over time [8]. The superinfection model extends the latter to the scenario where there are different states (or levels) of seropositivity resulting from recurrent malaria exposure [9]. However, this more complicated model does not have a dramatic impact on SCR estimation [9, 10] and, therefore, most likely to be excluded from routine data analysis.

The first step of a sero-epidemiological analysis invariantly assumes a constant SCR that applies to every individual in the population at all times. This assumption implies a simple and increasing SP curve taken as function of the age of the sampled individuals. However, there are several studies reporting a qualitative change of the SP at a given age value in relation to what is expected from a constant SCR assumption [7, 11, 12]. This change might result from more complex sero-epidemiological scenarios where SCR is assumed to vary over time or among distinct age groups, as reviewed elsewhere [13, 14]. Three main explanations were advanced for such qualitative change in SP, each one implying a different mathematical model to the data. Firstly, age-related behaviour might affect the malaria risk of certain age groups. An example of such risk behaviour was reported in Indonesia where SCR in adults was increased in relation to the SCR for younger individuals, most likely because of work-related activities in the forest and exposure to forest vectors [12, 15]. Secondly, a change in the SP curve could be related to putative founder effects, i.e., an influx of non-exposed migrants to an endemic region. Migrants and individuals born locally would have different infection history, thus, presenting different SP profiles. This situation occurred in Brazil where there was a wave of migration in the 1980’s from malaria-free states to mining sites in the heart of the Amazonia forest [16]. A similar founder effect was seen in Chagas disease in a Peruvian community [17]. Thirdly, a change in the SP curve might also be attributed to a reduction in malaria transmission after the implementation or intensification of a given malaria control programme [18]. It is expected that this scenario will become increasingly common and it is therefore important that surveys that collect serological information do so with sufficient statistical power to detect impact on malaria transmission and be informative for the control and research communities.

This paper focuses on sample size calculations for detecting an abrupt reduction in disease transmission occurred somewhere in the past. It is worth noting that this statistical exercise is affected by the transmission intensities acting before and after the reduction, and the time between the change point and sample collection. Until now only a few studies have reported a change in transmission using SP data and those referred to dramatic reductions in SCR after intervention (Table 1). Similar observation can be taken for sero-epidemiological studies of Chagas disease and trachoma [17, 19, 20]. One possible explanation is that a slowly decreasing trend in malaria transmission might not result in a clear qualitative change in the age-adjusted SP, as demonstrated in Western Kenya [21]. Alternatively it may be that studies were underpowered to detect small reductions in disease transmission.

**Table 1:** Reported seroconversion rate (SCR) estimates before and after a reduction in malaria transmission intensity at a given estimated change point (in years before sampling)

Country, site (antigen)	Sample size	SCR before^a^	SCR after^b^	Reduction (%)	Change point^b^
Bioko Island [10]					
Malabo (PfAMA1)	2181	0.17	0.05	70.6	4
North East (PfAMA1)	1171	0.16	0.03	81.3	6
South East (PfAMA1)	656	1.90	0.09	95.3	7
South West (PfAMA1)	568	0.72	0.07	90.3	12
Brazil [7]					
Anajás (PfAMA1/PfMSP1)	113	0.014	0.008	42.9	29
Jacareacanga (PfAMA1/PfMSP1)	172	0.514	0.017	96.7	29
Goianésia (PfAMA1/PfMSP1)	262	0.047	0.018	61.7	29
Itaituba (PfAMA1/PfMSP1)	183	0.014	0.004	71.4	29
Trairão (PfAMA1/PfMSP1)	204	0.024	0.011	54.2	29
Belém do Pará (PfAMA1/PfMSP1)	143	0.002	<0.001	>50	29
Tanzania [33]					
Same (PfMSP1)	1888	0.025	0.005	80.0	8
Same (PfAMA1)	1888	0.066	0.010	84.9	15
Vanuatu [15]					
Northern Tanna (PfMSP1)	361	0.060	0.012	80.0	32
Northern Tanna (PfSE)	362	0.051	0.006	88.2	32
Northern Tanna Highlands (PfSE)	514	0.045	0.005	88.9	27
Southern Tanna (PfSE)	364	0.012	0.001	91.7	18
Aneityum (PfSE)	517	0.015	0.001	93.3	23

^a^Expected number of events per year

^b^Years before sampling

Previously two sample size calculators were proposed for estimating SCR under stable disease transmission [22]. The present paper extends this work to the setting of detecting a reduction in SCR at a given time point before sampling and attributed to a field intervention. This new calculator is based on logistic approximations for the power using simulated data sets. As in a previous study [22], bias and precision of ensuing parameter estimates were also assessed via data simulation.

## Methods

### Reverse catalytic models for seropositivity data

Reverse catalytic models have recently been used to analyse seropositivity data [5, 13, 14]. Using a Markov chain formalism, these models describe the dynamics of the serological state of an individual under the assumption that every one is born seronegative and becomes seropositive upon malaria infection. Reversion to a seronegative state might occur in absence of sufficiently frequent malaria exposure. The basic notion is that the frequency by which individuals become seropositive (i.e., SCR) reflects the underlying force-of-infection while the rate by which seropositive individuals revert to a seronegative state (i.e., seroreversion rate (SRR)) might result from a variety of host factors (e.g., genetics or age).

The simplest epidemiological setting is to consider a constant disease transmission over time. In this situation, the resulting reverse catalytic model is described by the following probability of an individual aged *t* being seropositive.

1 $$ \pi_{t} = \frac{\lambda }{\lambda + \rho }\left(1 - e^{-(\lambda + \rho )t} \right) $$

where $\lambda$ and $\rho$ are SCR and SRR, respectively. Notwithstanding its simplicity, this model would appear to be appropriate for the *P. falciparum* malaria history of the Somalia [6], Brazilian Amazonian region [7], northeast Tanzania [8], or even when SCR randomly fluctuates around a given mean value over time [5].

A more interesting setting is to assume the occurrence of a sudden reduction in malaria transmission due to an intervention. In this scenario, SRR is commonly assumed to be constant over time, thus, precluding any significant changes in factors affecting seroreversion. The probability of an individual aged *t* being seropositive is now given by

2 $$ \pi_{t}^{*} = \left\{ {\begin{array}{*{20}l} {\frac{{\lambda_{2} }}{{\lambda_{2} + \rho }}\left( {1 - e^{{ - \left( {\lambda_{2} + \rho } \right)\tau }} } \right) + \frac{{\lambda_{1} }}{{\lambda_{1} + \rho }}\left( {1 - e^{{ - \left( {\lambda_{1} + \rho } \right)\left( {t - \tau } \right)}} } \right)e^{{ - \left( {\lambda_{2} + \rho } \right)\tau }} , \, } & {{\text{if }}t{ > }\tau } \\ {\frac{{\lambda_{2} }}{{\lambda_{2} + \rho }}\left( {1 - e^{{ - \left( {\lambda_{2} + \rho } \right)t}} } \right),} & {{\text{if }}t \le \tau } \\ \end{array} } \right. $$

where ${\lambda_1}\, \text {and} \,{\lambda_2}$ are the SCRs before and after the reduction in disease transmission, respectively $({\lambda_2} < {\lambda_1})\,\text{and}\, \tau$ is the time point when that reduction actually occurred (in years before sampling). In simple terms, the above equation is divided into two branches according to the time when the individuals were born in relation to the change time *t*. For individuals born before the change point (*t* > $\tau$), the seropositivity probability results from the sum of two probabilities: (1) one referring to the event of an individual being seropositive at the change point and remained so after that; and, (2) another one describing the chance of an individual being seronegative at the change point and seroconverting after that. The individuals born after the intervention (*t* < $\tau$) have only experienced current malaria transmission intensity and, thus, the corresponding seropositivity probability is described by the simple reverse catalytic model, as shown in Eq. (1). For a detailed mathematical derivation of the above equation, see Additional file 1.

### Model parameterization, estimation and comparison

Model parameterization was the same as previously described [22]. Briefly, the relationships between PR, entomological inoculation rate (EIR), SP, and SCR were used to derive realistic values for SCR and SRR. These relationships were derived from two independent data sets from northeast Tanzania where altitude is highly correlated to these different malaria risk measures. EIRs of 10, 1, 0.1, and 0.01 were used as the core values for studying reductions in disease transmission. The corresponding values for SCR associated with *P. falciparum* MSP1 antigen are 0.0969, 0.0324, 0.0108, 0.0036, respectively. Although some variations on SRR may be found across different studies, this parameter was fixed at 0.017 as the average value of a large study from 24 sites in Tanzania with differing malaria endemicity [5] and genetic background [23, 24].

With respect to parameter estimation, the sampling distribution was assumed to be a binomial-product distribution, one binomial distribution per age value. The simple reverse catalytic model was estimated via standard maximum likelihood method [25], whereas the parameter estimates of the model assuming a change in transmission were determined using a profile likelihood method. In general, the latter estimation method aims to reduce the dimensionality of the maximization problem associated with the maximum likelihood method. The basic idea is to maximize the log-likelihood function considering one of the parameters fixed at a given value. Maximization is successively carried out using different values of interest for that fixed parameter. The overall estimates are the ones associated with the maximum of all the maximized log-likelihood functions obtained from considering those fixed values. This simple idea has been applied to statistical problems where one aims to estimate a model that includes a parameter defined in the integer space, such as the number of different T cell receptors present in the organism [26]. In this line of thought, the change time point was then considered to be an integer value (i.e., years before sampling) leading to the following profile likelihood algorithm: (1) fix the change point $\tau$ at 1; (2) determine the respective maximum likelihood estimates for the remaining parameters; (3) calculate the corresponding log-likelihood function; (4) increase one unit (e.g., one year before sampling) to the change point and repeat steps (2-3); (v) keep increasing the change point until reaching the maximum expected value for that parameter. The final maximum likelihood estimates are those associated with the change point $\tau$ with the maximum value of the log-likelihood function. A detailed example on using this algorithm in real data can be found in Cook et al. [18].

After performing parameter estimation, the two reverse catalytic models were compared to each other using the Akaike’s information criteria (AIC) [27]. This pragmatic approach seems more appropriate than the popular Wilks’ likelihood ratio test because the usual Chi-Square approximation associated with the latter may be affected by the sample size to be determined. Theoretically, AIC weights the likelihood of a model given the data with its intrinsic dimension (i.e., the total number of parameters). One should then select the model that leads to the smallest AIC estimate. In this scenario, power to detect a reduction in SCR was estimated by the number of (simulated) data sets in which the corresponding model was considered better than the one assuming a stable SCR.

### Simulation study and sample size calculations

Before conducting the sample size calculation per se, data sets were first simulated from the simple reverse catalytic model assuming stable transmission intensity and tested against the reverse catalytic model but assuming a change in transmission. To simulate each data set, the following algorithm was used: randomly select the age of each individual in the sample, and then generate the seroposivity state of each individual at the time of sampling, using a Bernoulli trial with success probability given by the seroprevalence expected under a given model [22]. Assuming SRR = 0.017, four SCR situations were studied, 0.0969, 0.0324, 0.0108 and 0.0036, corresponding to 10, 1, 0.1 and 0.01 EIR units, respectively, as described elsewhere [22]. The total number of simulated data sets per SCR value was 1000 that seemed to provide a good precision of power estimates and feasible total time to perform the simulation study. The results demonstrated that sample sizes of at least 100 individuals ensure a null probability of detecting a spurious change point irrespective of the transmission intensity (Table 2). Therefore, given the typical sample sizes used in sero-epidemiological studies (see examples in Table 1), it is very unlikely to report a spurious change point.

**Table 2:** Percentage of simulated data sets detecting a change point where there is none using AIC for the comparison of the simple reverse catalytic model assuming stable transmission against the same model assuming a change in transmission

SCR (EIR)^a^	Sample size	AIC
0.0969 (10)	100	0.0
	250	0.0
	500	0.0
0.0324 (1)	100	0.0
	250	0.0
	500	0.0
0.0108 (0.1)	100	0.0
	250	0.0
	500	0.0
0.0036 (0.01)	100	0.0
	250	0.0
	500	0.0

^a^Expected number of events per year

**Fig. 1:**
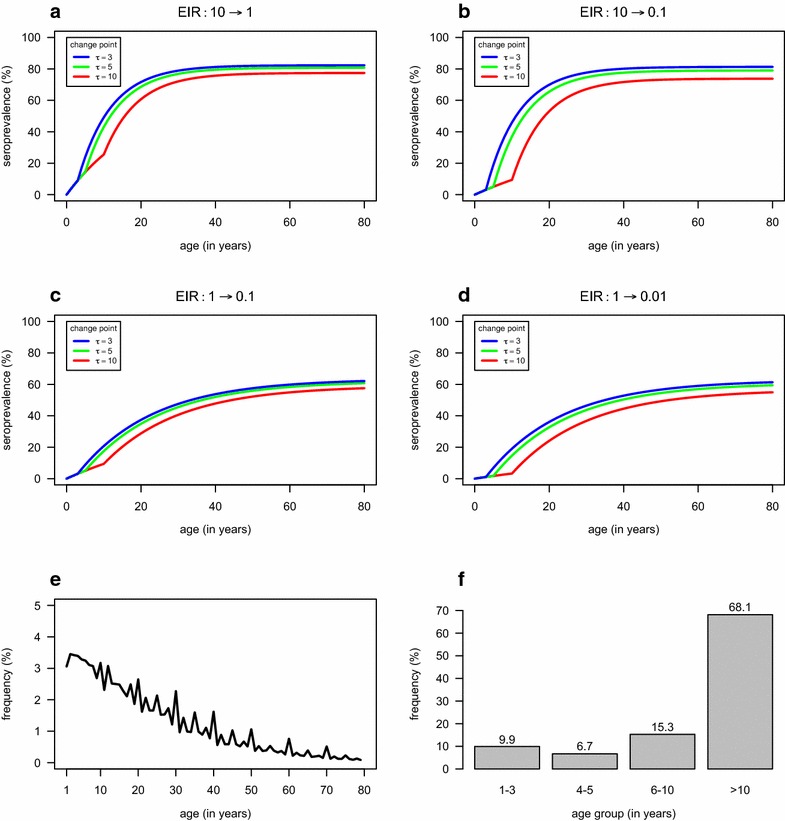
Key features of the simulation study. **a**–**d** Seroprevalence as expected by the indicated change in transmission intensity (as measured in EIR units) and the respective change point $\tau$. **e** Typical age distribution of a community survey from Africa; **f** Percentage of different age groups according to the age distribution shown in **e**

A simulation study under the assumption of a change in transmission was then performed using four different reductions in SCR: 0.0969 to 0.0324 and 0.0108 (10–1 and 0.1 in EIR units, respectively), and 0.0324 to 0.0108 and 0.0036 (1 to 0.1 and 0.01 in EIR units, respectively). These four reductions were then combined with three possible change points: three, five and ten years before sampling. In total, there are 12 parameter combinations under study that, in theory, comprise the most interesting situations for using a serological approach in malaria epidemiology. The corresponding age-adjusted SP curves are shown in Fig. 1a–d. At this point the visualization of these curves is key to obtain some qualitative expectation for the ensuing sample sizes. On the one hand, the reduction of one order of magnitude in EIR units does not show dramatic differences in the corresponding SP curves in relation to a situation of stable SCR (Fig. 1a, c), thus, implying larger sample sizes for the corresponding detection. On the other hand, the reduction of two orders of magnitude in EIR units shows a clear biphasic behaviour in the SP curves (Fig. 1b, d), thus relatively small sample sizes may be required, especially when those reductions occur ten years before sampling.

The total number of simulated data sets per parameter combination was 1000 assuming an appropriate balance between the precision of power estimates and the total time to perform the simulation study.

Central to the assumptions of the simulation study is the age distribution of a given population. For that a typical age distribution from African population was used (Fig. 1e), as described elsewhere [22]. To gain intuition on the relationship between the change point and the expected sample size, it was convenient to calculate the percentages of the following age groups: one to three, four to five, six to ten, >10 years old (Fig. 1f). These percentages imply that the frequency of individuals born after the reduction in transmission is 9.9, 16.6 and 31.9 % for the change points of three, five and ten years before sampling, respectively. This suggests that reductions in SCR occurring further in the past should be easier to detect than changes which occur closer to the time of sampling. Furthermore, since the frequency of the individuals born after the reduction increases with the change point, the precision of current SCR should also increase.

Before conducting any formal sample size calculation, the simulation results were first assessed for any potential bias of SCR and change point estimators. Although no sampling bias was introduced in the simulation of each data set, statistical theory predicts that the maximum likelihood method would only lead to unbiased estimates in settings of infinitely large samples [28]. The bias of a given estimator was estimated by the difference between the average of the estimates for a given parameter and the true parameter value that generated the data.

Approximate sample sizes were calculated by estimating power over a predefined set of sample sizes (e.g., 250, 500, 1000, 2500). The power was calculated under the assumption of a known and unknown change point. In some cases, simulation was extended to additional sample sizes (e.g., 100 or 5000) in order to increase the resolution of the underlying power curve. To approximate the power functions, separate logistic regression models were fit to the power estimates obtained from each one of the 12 parameter combinations; the package *easynls* for the R software was used for such purpose. In these models, the sample size was considered as a covariate. Better model fits were obtained using the sample size in log rather than in linear scale. The use of this transformed scale also ensured a null power for a sample size of 0, as predicted by statistical theory. The minimum sample size that would warrant a power $\beta_{0}$ was determined by the smallest integer greater than the value provided by the following formula

3 $$ n_{{i,\beta_{0} }} = \frac{{\log \left( {{{\left( {1 - \beta_{0} } \right)} \mathord{\left/ {\vphantom {{\left( {1 - \beta_{0} } \right)} {\beta_{0} }}} \right. \kern-0pt} {\beta_{0} }}} \right) - \hat{a}_{{i,\beta_{0} }} }}{{\hat{b}_{{i,\beta_{0} }} }}, $$

where $n_{{i,\beta_{0} }}$, $\hat{a}_{{i,\beta_{0} }}$ and $\hat{b}_{{i,\beta_{0} }}$ are the sample size, the intercept and the slope of the logistic regression estimated from simulated power associated with the *i*-th parameter combination (*i* = 1,…, 12), respectively. Sample sizes were calculated for $\beta_{0}$ = 0.80, 0.90 and 0.95.

The final step of this study was to learn the estimation implications of the calculated sample size. In particular, it was of key interest to assess the bias and relative precision associated with the estimates for current SCR. Bias was calculated as described above while estimation of relative precision was conducted as for the situation of stable SCR [22]. In brief, relative precision associated with a given sample size was defined as the difference between 2.5 and 97.5 % quantiles of the distribution of current-SCR estimates divided by the true parameter value that generated the corresponding data. This difference was calculated for the predefined set of sample sizes and then predicted for a specific one using the following linear regression model

4 $$ pr_{{n_{i} }} \left( {\hat{\lambda }_{2} } \right) = \hat{\gamma }_{0,i} + \frac{{\hat{\gamma }_{1,i} }}{{n_{i} }} + \frac{{\hat{\gamma }_{2i} }}{{n_{i}^{2} }} + \frac{{\hat{\gamma }_{3i} }}{{n_{i}^{3} }} $$

where $\hat{\gamma }_{0,i}$, $\hat{\gamma }_{1,i}$, $\hat{\gamma }_{2,i}$ and $\hat{\gamma }_{3,i}$ are coefficients estimated from the corresponding simulated data associated with *i*-th parameter combination. Further details on the use of this model for estimating precision can be found in a previous study [22].

All simulations and estimations were performed in the R software (version 3.2.1) using scripts written for the purpose. In a near future these scripts together with others for the analysis of stable transmission models will be assembled in a convenient R package. For now they are available from the first author upon request and free to be adapted to different sampling scenarios. It is worth noting that, to speed up the simulation study, parallel computing was carried out manually by running the analysis of each parameter in a different node of a computer cluster.

## Results

### Estimation bias when the true change point is assumed to be known and unknown

The simulated results were firstly studied in terms of estimation bias in relation to the true parameter values that generated the data (Table 3). When the simulated data sets were analysed assuming a known change point, the resulting SCR estimates showed slight bias for sample sizes of 250 and 500 individuals. Unsurprisingly, the most extreme case was observed for a change point of ten years before sampling and a reduction in SCR from 0.0324 to 0.0108 (from 1 to 0.1 in EIR units, respectively). For sample sizes of 1000 and 2500 individuals, estimation bias was highly reduced and tended to be close to the nominal value of 0 %. Since the change point was considered known and there was no selection bias introduced by a community-based sampling scheme, the estimation bias must be derived from the maximum likelihood method itself. Therefore, SCR estimation based on this method might require a bias-correction adjustment specifically for small sample sizes.

**Table 3:** Bias of SCR estimates before and after a change in transmission assuming the true change point known

			True time point for change in transmission					
			3 years^b^		5 years^b^		10 years^b^	
SCR before^a^	SCR after^a^	Sample size	SCR before	SCR after	SCR before	SCR after	SCR before	SCR after
0.0969	0.0324	250	7.6	−7.7	9.3	−3.6	15.9	−1.9
		500	2.5	−1.4	4.4	−0.4	8.1	0.1
		1000	1.4	−5.4	1.6	0.2	4.3	0.0
		2,500	0.4	1.0	1.4	0.0	1.1	−0.5
0.0969	0.0108	250	2.3	−6.7	5.9	1.1	11.1	−1.2
		500	1.0	0.8	4.4	−2.7	5.6	0.1
		1000	0.6	1.8	0.8	0.5	2.5	0.2
		2500	0.2	−0.3	1.0	−1.4	1.5	−0.4
0.0324	0.0108	250	6.9	−6.6	12.3	−5.6	36.5	−2.9
		500	5.3	−4.8	4.8	−3.2	13.2	−0.3
		1000	1.5	0.0	3.0	−1.7	5.0	−0.4
		2500	0.1	0.4	1.6	0.5	3.0	−0.1
0.0324	0.0108	250	4.1	11.0	8.5	−2.8	20.0	−1.5
		500	2.9	12.7	4.4	−6.6	7.1	0.5
		1000	1.4	−1.1	1.5	2.2	3.2	1.2
		2500	0.9	−1.7	0.8	−0.7	1.0	−0.3

^a^Expected number of events per year

^b^Years before sampling

**Fig. 2:**
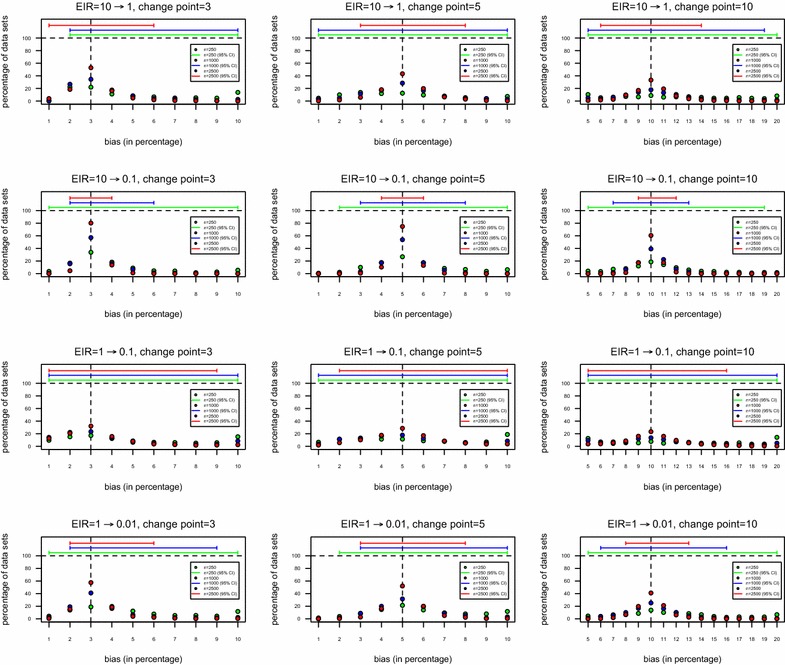
Distributions of the change point estimates according to the 12 parameter combinations used in the simulation study. For each parameter combination, the plot represents the variations in the distribution of change point estimates obtaining from 1000 simulated data sets with a given sample size using a profile likelihood method for the respective estimation. The corresponding 95 % confidence intervals for the true change point are shown on *top* of each plot

When the simulated samples were analysed under the assumption of a unknown change point, the SCR estimates were highly biased for sample sizes of 250, 500 and 1000 individuals (Table 4). In particular, the estimates of past SCR tended to overestimate the true parameter value whereas the opposite happened for current SCR where a negative bias was found for the corresponding estimates. Again, the most extreme estimation bias was observed for a change in transmission occurring ten years before sampling from 0.0324 to 0.0108 (1 to 0.1 in EIR units, respectively). Likewise for the case of a known change point, some estimation bias might result from the application of the maximum likelihood method itself. However, the highest contribution for estimation bias in this situation would appear to derive from highly skewed distributions for the change point estimates (Fig. 2 and Additional file 2). This skewness implied a tendency of overestimating the true change point and, because of that, estimation of the historic SCR might only use limited sample information of individuals likely to be in the plateau of age-adjusted SP curves (Fig. 1a–d). In practice, when overestimation of the change point occurs, the simple model assuming a constant SCR was mostly preferred to the data. Finally, it is worth noting the wide confidence intervals for the true change point even for sample sizes of 2500 individuals (Fig. 2, Additional file 2). This result suggests that the antibody data taken as a binary outcome might not have sufficient information to estimate the true change point with a high precision, thus, demonstrating the necessity of finding alternative approaches for that specific purpose. As an exceptional case, the situation related to a reduction from 0.0969 to 0.0108 (from 10 to 0.1 in EIR units) using a sample size of 2500 individuals implied at least 60 % chance of generating a data set that would lead to the correct change point estimate and relatively small confidence interval.

**Table 4:** Bias of SCR estimates before and after a change in transmission under the assumption of a unknown change point

			True time point for change in transmission						
			3 years^b^			5 years^b^		10 years^b^	
SCR before^a^	SCR after^a^	Sample size	SCR before	SCR after		SCR before	SCR after	SCR before	SCR after
0.0969	0.0324	250	368.2	−11.4		549	−23.2	1835.9	−7.1
		500	34.2	−22.1		47.4	−21.9	998.1	−2.4
		1000	11.2	−8.2		9.1	−10.7	143.1	−2.4
		2500	2.5	−5.1		4.4	−3.0	8.4	−1.6
0.0969	0.0108	250	215.5	−9.3		113.8	−17.6	671.3	−10.0
		500	18.0	−8.2		23.8	−17.0	96.6	−4.1
		1000	5.9	1.3		5.2	−3.4	27.3	−2.0
		2500	2.0	4.7		2.1	−2.6	3.2	−1.4
0.0324	0.0108	250	325.3	−14.0		191.9	−26.7	5384.0	−11.2
		500	66.0	−21.5		25.4	−32.8	1227.1	−7.8
		1000	13.2	−24.6		13.2	−25.2	126.7	−5.0
		2500	5.2	−16.6		6.4	−9.5	12.9	−2.8
0.0324	0.0108	250	534.7	20.9		105.9	−29.9	2254.6	−10.2
		500	59.8	0.6		31.0	−25.2	237.8	−9.4
		1000	12.6	−8.2		10.6	−11.3	20.8	−7.4
		2500	4.7	−0.4		4.5	−2.4	4.7	−3.2

^a^Expected number of events per year

^b^Years before sampling

### Sample size determination

**Fig. 3:**
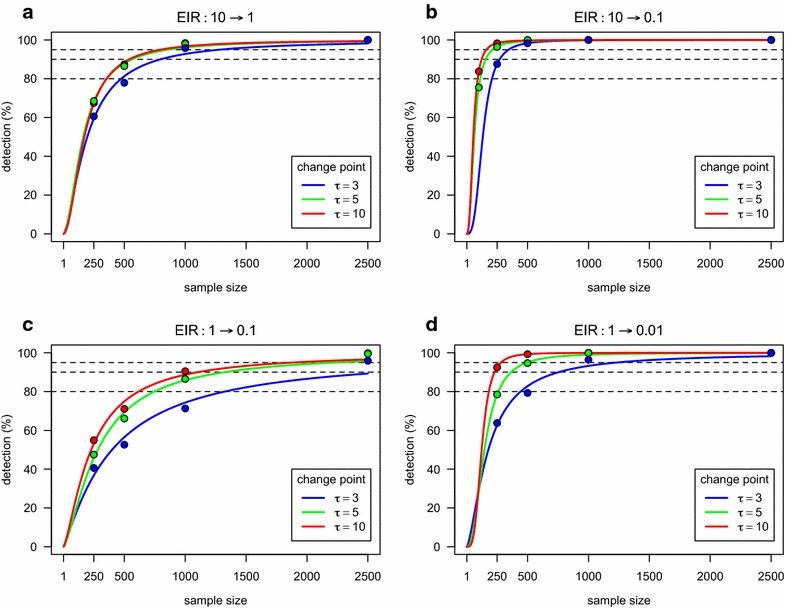
Statistical power to detect a change in SCR as function of sample size assuming the true change point is known. Changes in SCR are given in EIR units for better readability.* Solid lines* represent the best logistic function that could be fit to the respective simulated results (*filled circles*)

Sample size calculations were then performed using logistic curves fit to the simulated power (Fig. 3, Additional file 3). The sample size decreased with the true change point for a given value of power and reduction in disease transmission (Table 5). This implied that the detection of a short-term reduction requires larger sample sizes compared to settings where the same reduction is occurring further in the past. The exception would appear to be the analysis assuming a known change point and describing a reduction in SCR from 0.0969 to 0.0324 (Fig. 3a). In this case, the simulation results suggested that a reduction in SCR occurring five years before sampling was easier to detect than the same occurring ten years prior to sampling. However, the corresponding power functions were almost indistinguishable from each other and thus, these variations in the results might solely be attributed to the randomness associated with a simulation study. Notwithstanding these variations, it is clear that each parameter combination required a different set of sample sizes. On the one extreme, the lowest sample sizes were obtained for a reduction in SCR from 0.0969 to 0.0108 (from 10 to 0.1 in EIR units, respectively; Fig. 3b). This was unsurprising and agreed with the visual inspection of the SP curves shown in Fig. 1. In this case, a sample size of 485 individuals considering the change point known was enough to generate a power of at least 95 % to detect a reduction occurring between three and ten years before sampling. On the other extreme, the reduction in SCR from 0.0324 to 0.0108 (from 10 to 1 in EIR units, respectively; Fig. 3c) required the largest set of sample size irrespective of considering or not the change point known. The most extreme case was the sample size of 5675 individuals to detect a reduction occurring ten years before sampling with 95 % power under the assumption of a unknown change point.

**Table 5:** Minimum sample size to detect a change in transmission with 80, 90 and 95 % probability using AIC and under the assumption of a known or unknown true change point

			Assumption on the true change point					
			Known			Unknown		
SCR before^a^	SCR after^a^	True change point^b^	80 %	90 %	95 %	80 %	90 %	95 %
0.0969	0.324	3	475	797	1284	660	1158	1942
		5	356	562	857	441	707	1091
		10	355	541	798	445	742	1187
	0.0108	3	208	271	345	257	358	485
		5	112	156	212	148	215	304
		10	91	123	163	100	144	201
0.0324	0.0108	3	1324	2669	5091	1759	3236	5675
		5	748	1322	2234	997	1706	2798
		10	617	1104	1886	809	1513	2693
	0.0036	3	443	755	1234	695	1118	1731
		5	260	365	498	340	488	681
		10	182	229	283	219	275	339

^a^Expected number of events per year

^b^Years before sampling

### Estimation bias and precision associated with a given sample size calculation

Statistically speaking, the final decision of using of a given sample size must be taken not only on the basis of the underlying power, but also on the corresponding impact over parameter estimation. The above results suggested a negligible estimation bias for reasonably large sample sizes under the assumption of a known change point. In contrast, unbiased estimates might only be obtained for large sample sizes when the change point is considered unknown. Ideally, one wishes to have enough power to detect a reduction in SCR and high estimation precision.

For the analysis assuming a known change point, all determined sample sizes led to estimates for current SCR with up to 5 % bias (Table 6). The only exception was the setting of a reduction from 0.0969 to 0.0324 occurring three years before sampling where a -13 % bias was found for a power of 80 %. In this case, estimation bias can be avoided by increasing the power to 90 or 95 %. Although estimation bias was negligible for all determined sample sizes, the corresponding relative precision was in most cases greater than 1.00 in relation to the true value for current SCR. The only two settings where relative precision was less than 1.00 were related to reductions in EIR units of one order of magnitude for a change point of ten years before sampling. Therefore, when the change point is assumed to be known, sample size calculation based only on power, although avoiding estimation bias, would result in studies with limited estimation precision for current SCR.

**Table 6:** Expected bias and precision (in percentage) of estimates for current SCR using the sample sizes shown in Table 5

				Assumption on the true change point					
				Known			Unknown		
Estimation	SCR before^a^	SCR after^a^	True change point^b^	80 %	90 %	95 %	80 %	90 %	95 %
Bias^c^	0.0969	0.0324	3	−3.62	−2.18	−1.44	−17.15	10.16	−5.06
			5	−1.30	0.18	0.10	−23.62	−16.29	−10.27
			10	0.34	0.14	0.08	−5.20	−3.24	−2.39
		0.0108	3	−12.86	−4.85	−1.13	−13.95	−11.42	−7.91
			5	−3.20	−0.78	−0.04	−28.33	−23.17	−18.07
			10	0.23	−0.64	−0.88	−28.42	−23.02	−17.75
	0.0324	0.0108	3	−0.35	1.14	1.89	−19.30	−17.64	−16.67
			5	−2.10	−0.69	0.16	−22.70	−14.89	−9.85
			10	−0.25	−0.22	−0.34	−6.00	−3.96	−2.91
		0.0036	3	0.39	−4.13	−6.48	−0.37	−3.35	−4.96
			5	−2.82	−0.53	1.02	−27.64	−21.20	−16.04
			10	0.86	0.53	0.17	−17.20	−15.22	−13.42
Precision^d^	0.0969	0.0324	3	1.96	1.50	1.27	2.01	1.78	1.66
			5	1.48	1.21	1.06	1.65	1.65	1.46
			10	0.80	0.70	0.64	1.02	0.85	0.66
		0.0108	3	7.56	3.97	3.42	4.75	4.68	4.23
			5	3.62	3.22	3.10	3.13	3.13	3.05
			10	2.95	2.55	2.23	2.73	2.13	2.13
	0.0324	0.0108	3	2.04	1.74	1.55	2.10	1.84	1.68
			5	1.66	1.38	1.23	1.79	1.66	1.60
			10	1.01	0.83	0.73	1.40	1.04	0.80
		0.0036	3	5.23	4.43	3.97	4.82	4.14	3.73
			5	4.30	3.60	3.19	3.65	3.37	3.10
			10	2.70	2.70	2.58	2.96	2.95	2.86

^a^Expected number of events per year

^b^Years before sampling

^c^As percentage in relation to the true parameter value

^d^Difference between 2.5 and 97.5 % quantiles of the distribution of current−SCR estimates divided by the true parameter value that generated the corresponding data

For the analysis assuming a unknown change point, the sample size that would balance power, bias, and precision was not easily determined (Table 6). Most of the determined sample sizes led to underestimation with bias greater than 10 % in absolute terms, specially, when it was easy to detect a reduction in transmission (i.e., reduction in SCR of two orders of magnitude measured in EIR units). Having biased estimates would make any subsequent precision-based analysis elusive. Notwithstanding this problem, the corresponding results suggested poor estimation precision (>1.00) for the calculated sample sizes. Therefore, the uncertainty associated with a unknown change point brings problems in terms of balancing power with estimation bias.

## Discussion

This paper describes a pragmatic approach to calculate the minimum sample size for detecting a reduction in SCR with a given power. The approach was applied to different epidemiological settings but with a special focus on lower endemicity settings. The analysis was based on antibody responses to *P. falciparum* MSP1 antigens. A discussion about using antibody data from alternative antigens can be found elsewhere [22].

Sample size calculations were performed assuming demographics for African populations and using community-based surveys. The demographic distribution is different elsewhere and has been shown to impact SCR under stable SCR [22]. This study concluded that, under an unknown SRR, non-African studies using community sampling might require larger sample sizes than their African counterparts in order to obtain the same estimation precision. With respect to the present case of a reduction in SCR, one expects a higher precision of past-SCR estimates in non-African studies for a given sample size, owing to an increased frequency of older individuals that experienced both past and current malaria transmission intensity. In contrast, precision of current SCR would be increased in African studies due to a higher percentage of young individuals who only experienced current disease transmission intensity. Although expected, these estimation implications need to be further investigated. Alternatively, some statistical improvement might be achieved by sampling specific age groups. Malaria transmission due to *P. falciparum* is typically much lower outside Africa [29], indicating larger sample sizes for detecting putative reductions in SCR and the need for alternative sampling approaches. This is particularly important when transmission risk is behavioural and associated with older ages [12, 15].

Besides describing a framework for sample size calculation, this paper has also important implications in terms of estimation bias and precision, especially when the true change point is assumed to be unknown. In community surveys, the expected 95 % confidence intervals for the true change point tend to be wide suggesting a high uncertainty in estimating such parameter. The respective point estimates tend to overestimate the true change point being located further in the past than in reality. In agreement with this result is the serological study from the Bioko Island in Equatorial Guinea [11] where a cross-sectional survey was conducted four years after the initiation of a comprehensive malaria control programme in the island but the estimated change points suggested a reduction in transmission further in the past (Table 1). Similarly, a serological study from Vanuatu estimated a change point occurring 30 years before sampling that appeared to overestimate in 13 years a putative change point due to a known insecticide-treated net distribution across the islands (Table 1) [18]. Biologically speaking, the most likely explanation for obtaining overestimates of the change point is the putative difference in antibody-decay rates between younger children and older individuals. More precisely, the former would have a higher loss rates than the latter, who have more established antibody responses [30, 31]. In practice, it is unlikely to have enough information to distinguish between statistical and biological bias, thus the necessity of applying different bias-reduction strategies to data collection and analysis.

To minimize the estimation bias and decrease uncertainty of the estimates, five possible solutions can be envisioned. The first one consists of fixing the change point at an expected value. In that case, the SCR estimates were found to be approximately unbiased for relatively small sample sizes (e.g., 250 or 500 individuals depending on the size of the underlying reduction in SCR). Assuming a given change point would appear to be a reasonable data analysis strategy for post-intervention studies where the start of the intervention is known, such as the above-mentioned study from Bioko where an intensive malaria control programme was launched in 2004 [11]. However, fixing a change point might not be so easily applicable to exploratory (or preliminary) studies. As an example, a recent study from the Brazilian Amazonia region reported a strong reduction in SCR for *P. falciparum* antigens occurring 30 years before sampling [7]. In this specific example, different malaria control programmes have been operating in the area since the 1980’s [32] but are likely to have been scaled up over time making a known change point difficult to assess. Health system records of changes in malaria case number could additionally provide indicators of potential change points for a given study. The second solution for reducing bias is to use alternative estimators, such as the jackknife [33] or the bootstrap estimator [34], which are particularly tailored to solve this statistical problem. However, these estimators are in general computationally intensive due to the use of leave-one-out or re-sampling techniques. In this scenario, the application of these estimators would appear to be feasible in small samples where estimates might be more affected by bias. For large samples, estimation bias is reduced and, therefore, the decision of using such estimators should be weighted with the real implications of obtaining more reliable estimates. A third route for bias reduction is to choose a sampling strategy where the chance of detecting the true change point is increased. One might define three age groups according to the kind of epidemiological information each one provides. The first one refers to individuals born before the change point but with age ‘‘far’’ from it (e.g., young children up to three years old for a change point of five years before sampling). This age group is essential to estimate current SCR since the corresponding target population would not have experienced any change in disease transmission. The second group consists of children or adolescents with age in the vicinity of the putative change point (e.g., children aged between four to seven years old for a change point of five years before sampling), thus, having the highest sampling information over that parameter. The age range should be defined in order to approximately sample the same amount of individuals that experienced the different exposure periods. Sampling in this way should jointly increase the power to detect a change in SCR and the accuracy of the corresponding change-point estimates. The third group targets older individuals because of the putative information they might show of historical disease exposure. Since this group refers to older individuals, it also embodies important information on seroreversion rate. Having all of these different possible sampling options, future research is critical to determine the most optimal sampling strategy for controlling power, precision and bias altogether. A fourth solution is to jointly analyse data from different study sites. The theoretical expectation is that, under the assumption of a shared change point, more accurate information can be borrowed from sites where such a parameter is more easily estimated. This solution was followed in the above-mentioned Brazilian study where there is evidence for a common change point for *P. falciparum* malaria that could not be easily detected due to the uncertainty of the change point estimates when the corresponding data of each study site was analysed separately (Table 1) [7]. A fifth and last solution is to analyse antibody concentration data using appropriate antibody density models as reviewed elsewhere [13]. The use of a quantitative outcome is expected to be more informative about the underlying phenomenon than its binary-derived counterpart, as suggested by several genetic studies aiming to estimate the location of quantitative trait loci [35, 36]. Similar line of evidence was observed in a sero-epidemiological study from Nigeria where the antibody values of the sampled individuals declined over an intervention period but the corresponding age-adjusted seroprevalence curves remained unaltered [37, 38]. However, this solution remains to be tested in real world data.

In general, controlling power via sample size is an ideal strategy to increase the chance of drawing the right conclusion if different explanations exist for the same data. Here, the power was calculated using the stable SCR assumption as the only alternative explanation for the data. However, an effect of age-dependent risk factor might be yet another competing explanation for the occurrence of biphasic SP curves. This is the case of an Indonesian population where adults working in the forest were more exposed to malaria vectors than younger workers [15]. In theory, the intervention-based and risk-factor models are mathematically distinguishable but very closely related [13]. Therefore, the sample sizes calculated here would also hold for the alternative setting of detecting changes in SCR due to age-dependent risk factors, although this requires further investigation. Alternatively, the power to distinguish these models might require collecting such a large sample that brings several practical and theoretical challenges, as discussed in detail elsewhere [22]. Current models may be inappropriate in these settings and approaches that use antibody levels or different antigenic targets with shorter SRR would ultimately be more useful.

The calculated sample sizes suggest potentially opposing conclusions for study design. On the one hand, small sample sizes might be sufficient to detect significant reductions in SCR with high power, but lead to relatively poor estimation precision of current SCR. In this scenario, it is recommended to perform sample size calculations focusing on estimation precision rather than on power. Assuming the true change point known improves the estimates precision, which might be further improved by fixing the SRR at a reasonable value [22]. On the other hand, subtle reductions in SCR might only be detected by means of large sample sizes. The use of a large sample size brings theoretical and operational challenges but inevitably leads to improved estimation precision and reduced estimation bias. Precision and bias are particularly important to be controlled in situations where there is no information on the timing of a change in transmission.

## Conclusion

In summary, designing a study that aims to detect a reduction in transmission using SCR requires balancing the use of a given sampling strategy with the sample size warranting a given power and estimation precision. Ultimately the decision of choosing one or another sample size should be made on the basis of not only statistical arguments, as discussed here, but also on possible sampling constraints that might influence data collection, such as ethics, available human and economic resources and/or presence of any time constraint. Simply augmenting the number of individuals sampled in the age groups around any perceived change point may be the most pragmatic solution. As malaria transmission decreases and multiple malariometrics are required to determine the effect of control programmes, optimizing sample size is crucial to avoid wasting valuable resources. Using optimal study designs is particularly important for countries on the brink of malaria elimination or eradication, such as the Hispaniola Island [39] or Sri Lanka [40]. This and other related issues are going to be investigated in a future study.

## Additional files

10.1186/s12936-015-1050-3 Mathematical derivation of the reverse catalytic model assuming a change in transmission.
10.1186/s12936-015-1050-3 Distribution of the change point estimates as described in Fig. 2 but using the percentage of +1/−1 year unit in relation to the true change point.
10.1186/s12936-015-1050-3 Statistical power to detect a change in disease transmission as function of sample size considering the true change point unknown. See Fig. 3 for further details.

## Abbreviations

AIC: Akaike’s information criterion
EIR: entomological inoculation rate
SP: seroprevalence
SCR: seroconversion rate
SRR: seroreversion rate
AMA1: apical membrane protein-1
MSP1: merozoite surface protein-1
PR: parasite rate
