# Supplementary material for: Sample size and power calculations for detecting changes in malaria transmission using antibody seroconversion rate
Source: Malar J. 2015 Dec 30;14:529. doi: 10.1186/s12936-015-1050-3 (PMC4696297; doi:10.1186/s12936-015-1050-3)
Supplement: Supplementary file 1 — 10.1186/s12936-015-1050-3 Mathematical derivation of the reverse catalytic model assuming a change in transmission. [file 12936_2015_1050_MOESM1_ESM.pdf]

Additional file 1

Mathematical derivation of the reverse catalytic model assuming a change transmission

In this additional file, the reverse catalytic model assuming a change transmission is derived using a Markov chain formalism; see Kulkarni [1] for a general discussion about this mathematical modelling approach. As mentioned in the main text, the reverse catalytic model describes a stochastic process of two serological states that evolve randomly in time upon malaria exposure and in absence of it. The historical and current seroconversion rates are denoted by λ_1 and λ_2, respectively. The seroreversion rate ρ is considered constant over time as typically assumed in sero-epidemiological studies. The change point τ is described in relation to the time of data collection.

In this setting, one needs to distinguish the dynamics of individuals born before and after the change point. On the one hand, an individual with age $t < \tau$ (born after the change point) did not experience any change transmission in disease transmission and, therefore, the expected seroprevalence is given by the simple reverse catalytic assuming the current seroconversion rate, i.e.,

$$\pi_t^+ = \theta_2 \left(1 - e^{-\gamma_2 t}\right), \tag{1}$$

where $\theta_2 = \lambda_2/(\lambda_2 + \rho)$ and $\gamma_2 = \lambda_2 + \rho$. On the other hand, an individual with age $t > \tau$ (born before the change point) has experienced $t - \tau$ and τ years of the historical and current seroconversion rates, respectively. In this case, an individual has the following probabilities of being seronegative and seropositive at age $t - \tau$ (end of the first time period)

$$\pi_{1,t-\tau}^- = 1 - \theta_1 \left(1 - e^{-\gamma_1 (t-\tau)}\right) \tag{2}$$

and

$$\pi_{1,t-\tau}^+ = \theta_1 \left(1 - e^{-\gamma_1 (t-\tau)}\right). \tag{3}$$

where $\theta_1 = \lambda_1/(\lambda_1 + \rho)$ and $\gamma_1 = \lambda_1 + \rho$. Then the seroconversion rate is abruptly reduced from λ_1 to λ_2. The following stochastic process is modelled again by the simple reverse catalytic model with the difference of starting with initial probabilities as described below.

In its simplest formulation, the reverse catalytic model can be seen as a Markov chain as described elsewhere [2]. Under this idea, the Markov chain associated with the second time period is first described by the following transition rate matrix

$$R = \begin{bmatrix} -\lambda_2 & \lambda_2 \\ \rho & -\rho \end{bmatrix}. \tag{4}$$

In general, the transient behaviour of a Markov chain can be written as the following first-order stochastic differential equation

$$P'(t) = P(t)R \tag{5}$$

where $P(\tau)$ is the so-called transition matrix with each entry (i, j) describing the conditional probability of an individual being in state j at an arbitrary time point t given that individual started the process in state i. The solution of the above stochastic differential equation is simply given by

$$P(t) = e^{Rt}. \tag{6}$$

For the model at hand and a time period of length τ, the above equation converts into

$$e^{R\tau} = \begin{bmatrix} 1-\theta_2(1-e^{-\gamma_2\tau}) & \theta_2\left(1-e^{-\gamma_2\tau}\right) \\ \\ (1-\theta_2)\left(1-e^{-\gamma_2\tau}\right) & \theta_2+(1-\theta_2)e^{-\gamma_2\tau} \end{bmatrix}, \tag{7}$$

where R is given by equation (4). Since the individuals born before the change point start the second time period with initial probability vector p_0 given by equations (2) and (3) (seronegative and seropositive probabilities at the end of the first time period), the overall probabilities π_t^- and π_t^+ of seronegativity or seropositivity at the time of data collection (e.g., at age t) can be expressed as follows

$$\begin{bmatrix} \pi_t^- \\ \pi_t^+ \end{bmatrix} = p_0 e^{Rt} = \begin{bmatrix} 1-\theta_2\left(1-e^{-\gamma_2\tau}\right)-\theta_1\left(1-e^{-\gamma_1(t-\tau)}\right)e^{-\gamma_2\tau} \\ \theta_2\left(1-e^{-\gamma_2\tau}\right)+\theta_1\left(1-e^{-\gamma_1(t-\tau)}\right)e^{-\gamma_2\tau} \end{bmatrix}. \tag{8}$$

References

[1] Kulkarni, Vidyadhar G. Modeling and analysis of stochastic systems. CRC Press, 2009.

[2] Sepúlveda N, Drakeley C. Sample size determination for estimating antibody seroconversion rate under stable malaria transmission intensity. Malar J. 2015, 14:141.
