# Supplementary figures and images for: Sample size and power calculations for detecting changes in malaria transmission using antibody seroconversion rate
Source: Malar J. 2015 Dec 30;14:529. doi: 10.1186/s12936-015-1050-3 (PMC4696297; doi:10.1186/s12936-015-1050-3)

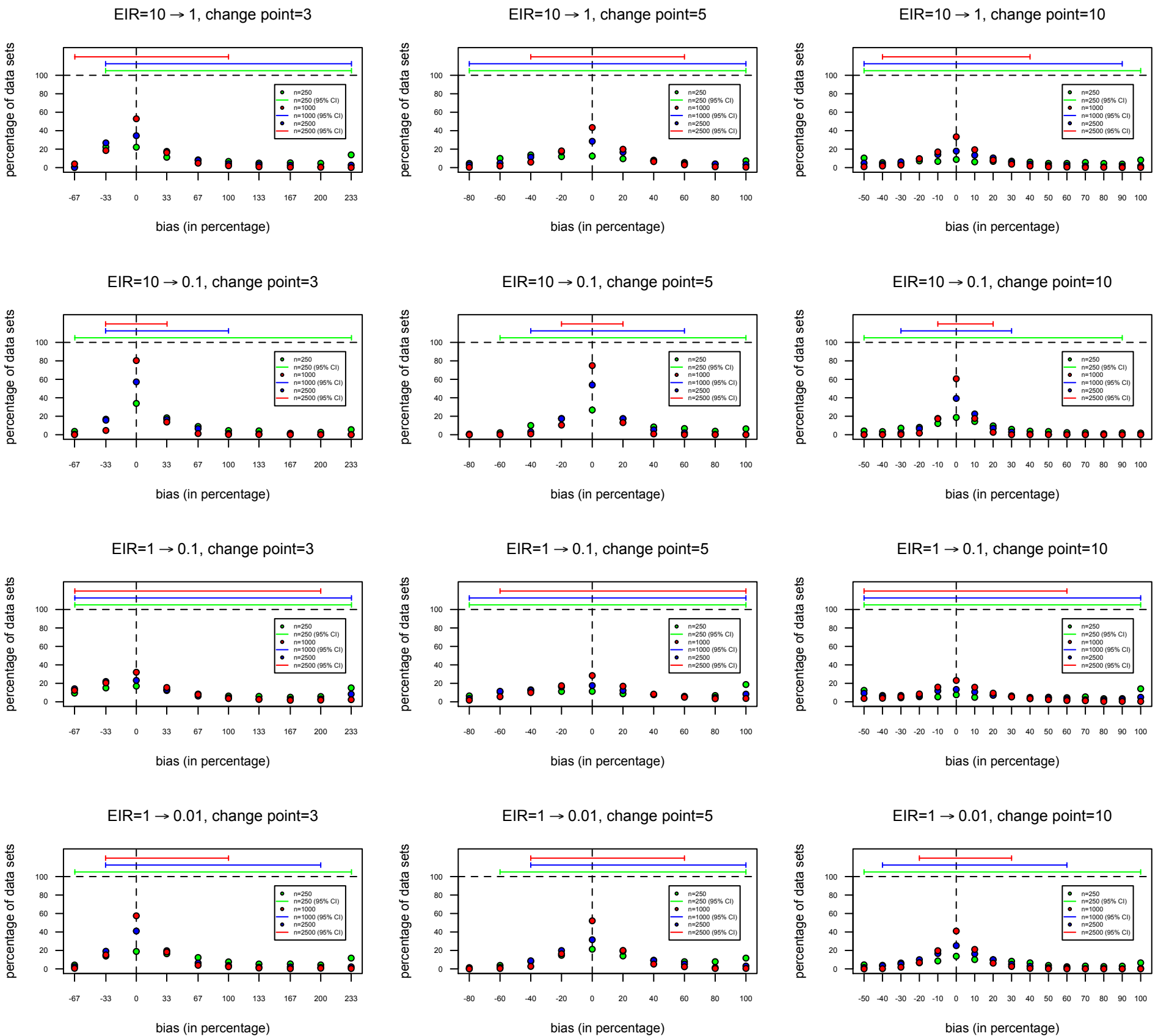

Supplement: Supplementary file 2 — 10.1186/s12936-015-1050-3 Distribution of the change point estimates as described in Fig. 2 but using the percentage of +1/−1 year unit in relation to the true change point. [file 12936_2015_1050_MOESM2_ESM.pdf]

**A**EIR :  $10 \rightarrow 1$ 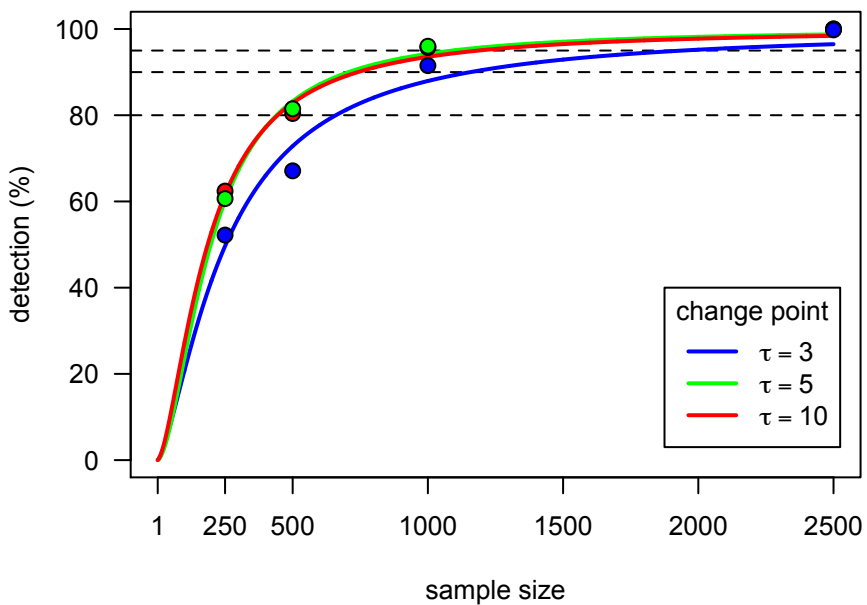**B**EIR :  $10 \rightarrow 0.1$ 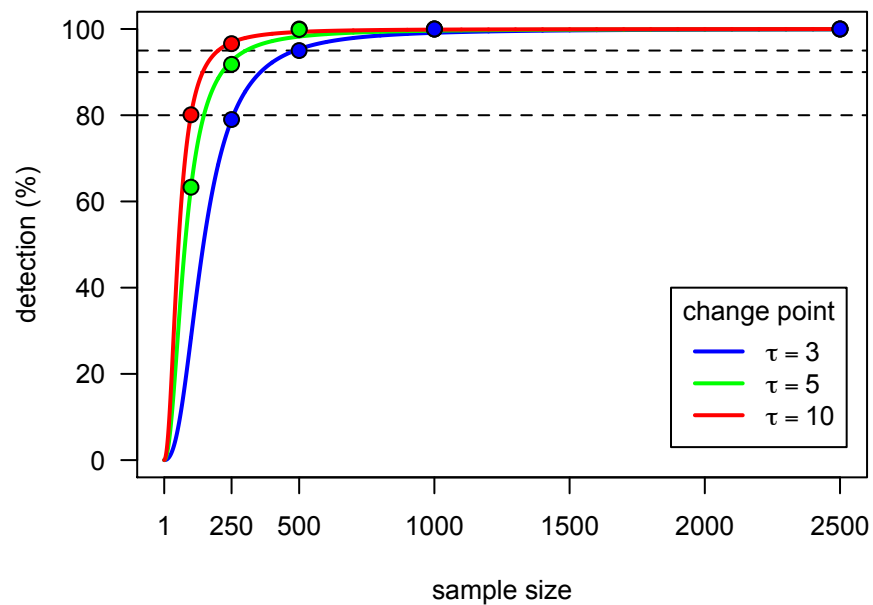**C**EIR :  $1 \rightarrow 0.1$ 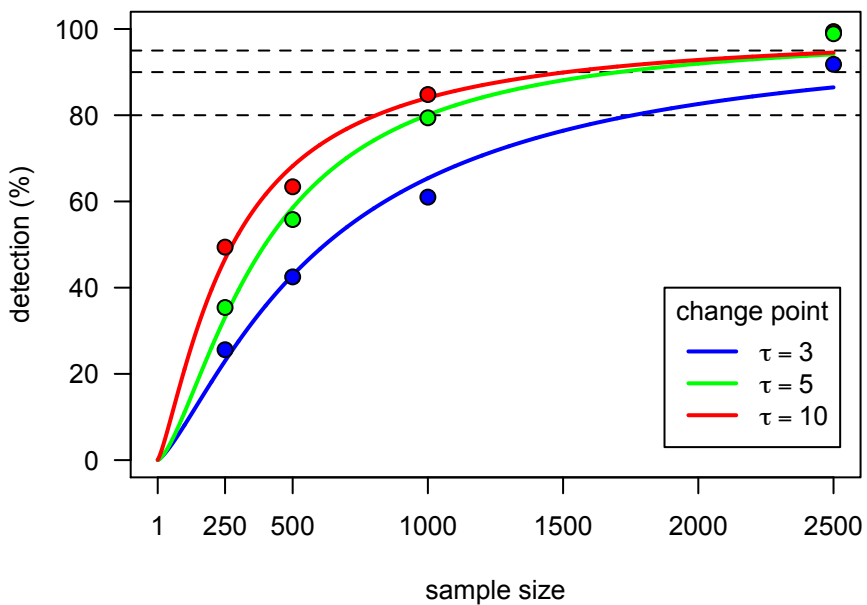**D**EIR :  $1 \rightarrow 0.01$ 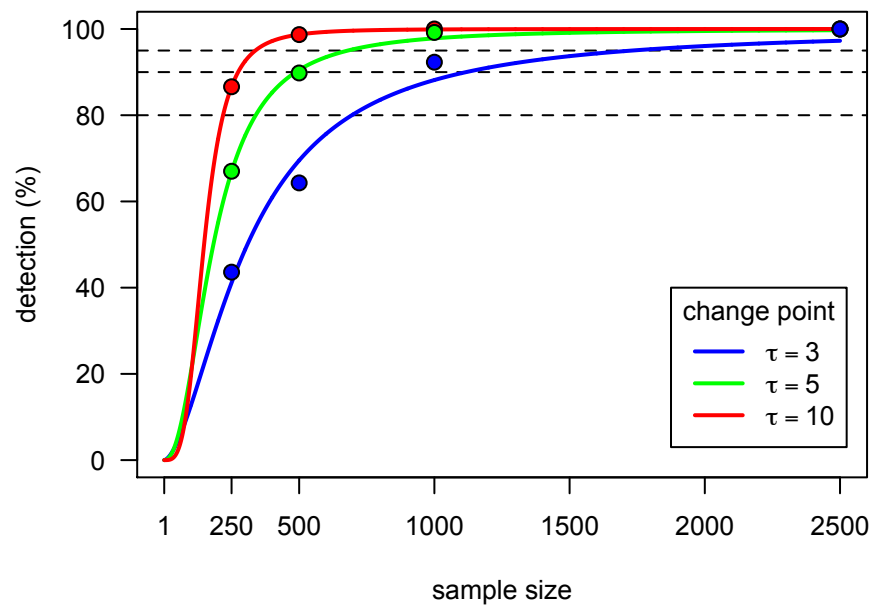

Supplement: Supplementary file 3 — 10.1186/s12936-015-1050-3 Statistical power to detect a change in disease transmission as function of sample size considering the true change point unknown. See Fig. 3 for further details. [file 12936_2015_1050_MOESM3_ESM.pdf]
